# Seasonal patterns in nest survival of a subtropical wading bird, the Hawaiian Stilt (*Himantopus mexicanus knudseni*)

**DOI:** 10.7717/peerj.10399

**Published:** 2021-02-01

**Authors:** Kristen C. Harmon, Nathaniel H. Wehr, Melissa R. Price

**Affiliations:** Department of Natural Resources & Environmental Management, University of Hawai‘i at Mānoa, Honolulu, HI, USA

**Keywords:** Seasonality, Introduced predators, Nest survival, Vegetation, Proximity to water

## Abstract

Nest survival is influenced by where and when birds decide to breed. For ground-nesting species, nest-site characteristics, such as vegetation height and proximity to water, may impact the likelihood of nest flooding or depredation. Further, habitat characteristics, and thus nest survival, may fluctuate across the breeding season. The Hawaiian Stilt (‘Ae‘o; *Himantopus mexicanus knudseni*) is an endangered Hawaiian waterbird that nests in wetlands across the Hawaiian Islands. In this study, we used observational surveys and nest cameras to examine the impact of nest-site characteristics and day of nesting season on nest survival of the Hawaiian Stilt. Early nests had a higher chance of survival than late nests. For most of the nesting season, taller vegetation was correlated with increased nest survival, while shorter vegetation was correlated with increased nest survival late in the nesting season. Seasonal patterns in nest survival may be due to changes in parental behavior or predator activity. Nest depredation was responsible for 55% of confirmed nest failures and introduced mammals were the primary nest predators. Our study is the first to examine seasonality in nest survival of Hawaiian Stilts and suggests that, despite longer nesting seasons and year-round occupation of wetlands, late nesters in subtropical regions may have lower nest survival than early nesters, similar to trends observed in temperate regions.

## Introduction

Where and when birds decide to breed greatly impacts nest survival (Sanchez-Lafuente, Alcántra & Romero, 1998; Golawski & Mitrus, 2008). Nest-site characteristics, such as vegetation height and proximity to water, impact nest survival by affecting the likelihood of nest flooding (Desgranges et al., 2006) or nest depredation (Jedlikowski, Brzeziski & Chibowski, 2015; Hoover, 2006), particularly for ground-nesting species (Lima, 2009). Further, nest survival is often not constant across the breeding season (Wilson, Martin & Hannon, 2007; Polak, 2016) and may vary due to changes in habitat conditions (Polak & Kasprzykowski, 2013) or depredation pressure associated with nest density (Nams, 1997; Elmberg et al., 2009) or parental activity (Skutch, 1949; Martin, Scott & Menge, 2000). Studies of shorebirds in temperate regions show that early-season nests typically have higher nesting success than late-season nests (Thyen & Exo, 2005; Cuervo, 2010; Jakubas, 2011). However, few studies have examined seasonality in nest survival in tropical or subtropical regions (Morrison, 1999; Olmos & Silva, 2001), and even fewer have been conducted on tropical or subtropical shorebirds (Ramos, 2001).

The Hawaiian Stilt (‘Ae‘o; *Himantopus mexicanus knudseni*), a federally endangered subspecies of the Black-necked Stilt, inhabits freshwater and brackish wetlands across the Hawaiian Islands (U.S. Fish & Wildlife Service, 2011). Hawaiian Stilts are semi-colonial nesters, and nests are placed on the ground, typically in low-lying vegetation or in scrapes on mudflats, with an average clutch size of 3–4 eggs (Coleman, 1981). Eggs are typically incubated for 24–26 days, and chicks are precocial, leaving the nesting area within hours of hatching (Coleman, 1981). Prior research on Hawaiian Stilts has primarily focused on life history, population viability, and movement (Reed et al., 1998, 1999, 2014; Kawasaki, Hart & Paxton, 2019), with few studies examining factors that impact reproductive success (Coleman, 1981; van Rees & Reed, 2020). Hawaiian Stilts are threatened by a variety of mammalian, avian, and aquatic predators, including introduced species such as rats (*Rattus spp*.), feral cats (*Felis catus*), Small Indian Mongooses (*Herpestes auropunctatus*), Cattle Egrets (*Bubulcus ibis*), Barn Owls (*Tyto alba*), Catfish (Order: *Siluriformes*), Cane Toads (*Rhinella marina*), and American Bullfrogs (*Lithobates catesbeianus*), as well as native Black-crowned Night-Herons (‘Auku‘u; *Nycticorax nycticorax hoactli*) and Hawaiian Short-eared Owls (Pueo; *Asio flammeus sandwichensis*) (U.S. Fish & Wildlife Service, 2011). Nest depredation and flooding have been suggested as major threats to Hawaiian Stilt nesting success (Coleman, 1981; U.S. Fish & Wildlife Service, 2011), and as such, nest-site characteristics, such as vegetation height and proximity to water, may impact nest survival.

Compared to Black-necked Stilts in continental systems, which nest from April to August in temperate regions (Carmona et al., 2000; Conway, Smith & Ray, 2005; Ackerman et al., 2014), Hawaiian Stilts are capable of nesting year round, although peak nesting typically takes place from March to August (Coleman, 1981). Increased nesting opportunities due to a prolonged breeding season suggest within-season timing of nesting may not be as important for determining nesting success of Hawaiian Stilts, as is demonstrated in other stilt species in temperate regions (Cuervo, 2010; Ackerman et al., 2014). However, precipitation on most islands in Hawai‘i varies temporally, with the “wet season” occurring October through April, and the “dry season” May through September (Price, 1983). The Hawaiian Stilt nesting season begins during the “wet season” and coincides with a seasonal decline in rainfall across the islands, which may cause temporal changes in nest vegetation height or proximity to water, leading to differences in nest survival. To better understand Hawaiian Stilts in comparison to closely related continental species, the aim of this study was to determine the impact of seasonality and nest-site characteristics on nest survival.

## Materials and Methods

### Study site

**Figure 1:**
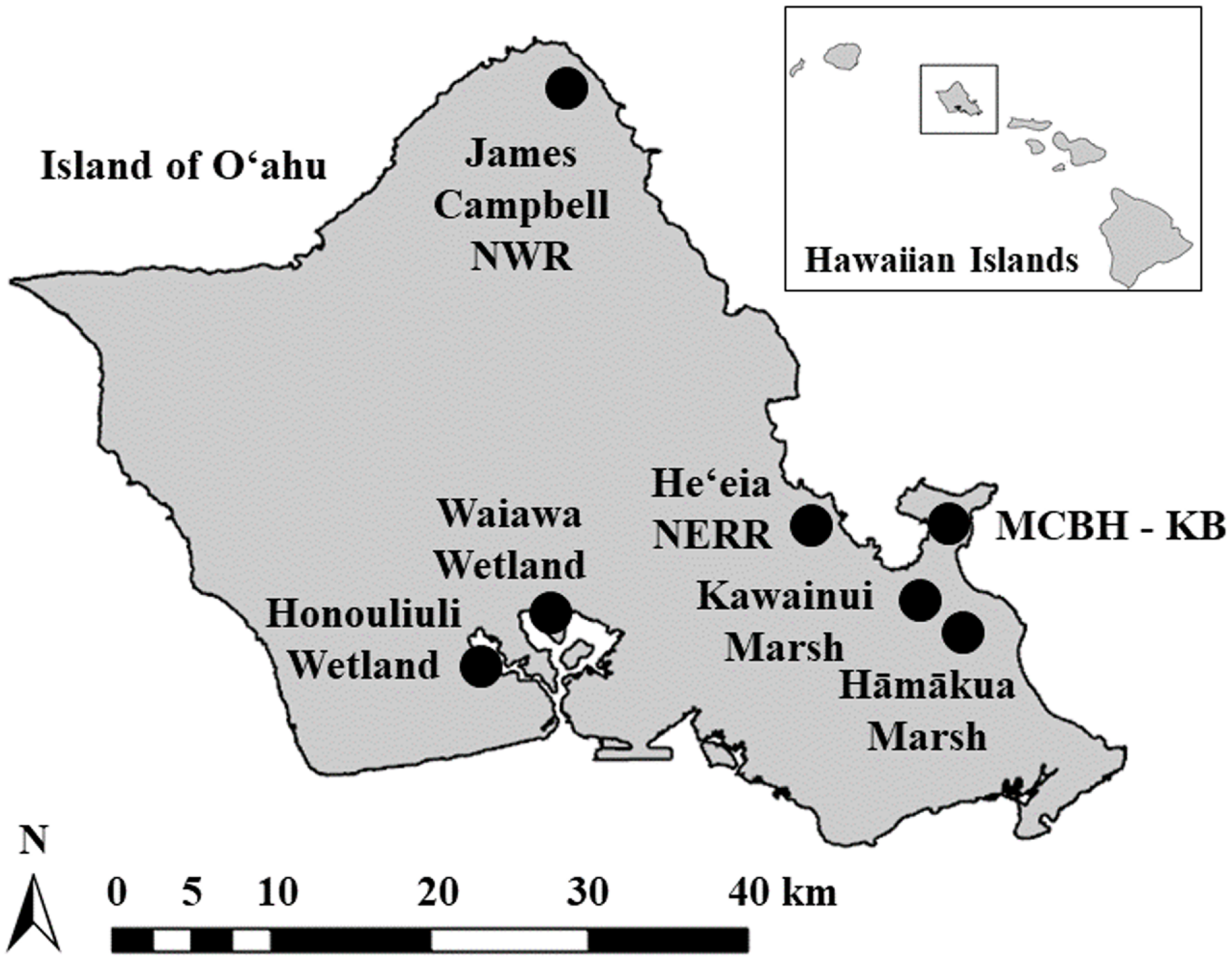
Island of O‘ahu, Hawai‘i, USA. Study sites, indicated by shaded circles, included Hāmākua Marsh, Kawainui Marsh, Marine Corps Base Hawaii—Kaneohe Bay (MCBH-KB), Honouliuli Wetland, Waiawa Wetland, James Campbell National Wildlife Refuge (NWR), and He‘eia National Estuarine Research Reserve (NERR).

This study was conducted in seven wetlands on the island of O‘ahu, Hawai‘i, USA (Fig. 1; Table 1). Kawainui Marsh, located on the windward (east) side of O‘ahu, is the largest wetland in the state of Hawai‘i and was designated a Ramsar Wetland of International Importance in 2005. Kawainui includes 11 human-made, shallow, freshwater ponds that make up ~16 ha out of the 300-ha marsh. During large precipitation events the ponds fill with water by direct rainfall, as well as overflow from an adjacent stream, and may be drained by land managers during outbreaks of avian botulism (U.S. Fish & Wildlife Service, 2011). Hāmākua Marsh, also located on the windward side of O‘ahu, is a brackish wetland that is ~8 ha. Water levels in Hāmākua fluctuate according to direct rainfall, runoff from surrounding land, and occasional managed tidal inundation. Kawainui and Hāmākua are managed by the Hawai‘i State Department of Land and Natural Resources.

**Table 1:** Table summarizing study site attributes. Study sites included Hāmākua Marsh, Kawainui Marsh, Marine Corps Base Hawaii—Kaneohe Bay (MCBH-KB), Honouliuli Wetland, Waiawa Wetland, James Campbell National Wildlife Refuge (JCNWR), and He‘eia National Estuarine Research Reserve. Coordinates for each site represent the starting location of nest surveys in each wetland. Sites are managed either by the Hawai‘i Department of Land and Natural Resources (DLNR), MCBH-KB, the U.S. Fish and Wildlife Service (USFWS), or the Hawai‘i Community Development Authority (HCDA). Sites receive different sources of water, such as direct rainfall, runoff, stream input, and tidal fluctuations, as well as different degrees of water depth management, such as pumping water from wells, diverting streams, and draining. Most sites manage invasive vegetation via mowing. The MCBH-KB uses amphibious assault vehicles (AAV) to remove invasive vegetation and create mudflat habitat, while also providing training to military personnel. At He‘eia, in addition to mowing, hand weeding and sheep grazing are used to control invasive vegetation.

Site	Year(s) surveyed	Coordinates	Size (ha)	Managing entity	Water source	Water depth management	Invasive vegetation management
Hāmākua	2018, 2019	21.3866°N, 157.7388°W	8	DLNR	Rainfall, runoff, tidal	Managed tidal flooding, draining	Mowing
Kawainui	2018, 2019	21.3827°N, 157.7593°W	16	DLNR	Rainfall, stream	Draining	Mowing
MCBH-KB	2019, 2020	21.4287°N, 157.7436°W	60	MCBH-KB	Rainfall, tidal	None	AAV’s
Honouliuli	2018, 2019	21.3567°N, 158.0210°W	10	USFWS	Rainfall, well water	Well water pumping, draining	Mowing
Waiawa	2018, 2019	21.3882°N, 157.9819°W	15	USFWS	Rainfall, well water	Well water pumping, draining	Mowing
JCNWR	2018, 2019	21.6844°N, 157.9543°W	51	USFWS	Rainfall, stream, tidal	Well water pumping, draining	Mowing
He‘eia	2019	21.4339°N, 157.8112°W	120	HCDA	Rainfall, stream	Stream diversions, draining	Mowing, hand weeding, sheep grazing

The Marine Corps Base Hawaii—Kaneohe Bay (MCBH-KB) is also located on the windward side of O‘ahu. Hawaiian Stilts at the MCBH-KB primarily utilize the Nu‘upia Ponds Wildlife Management Area, the Salvage Yard wetland, Sag Harbor wetland, Hale Koa wetland, and the water reclamation facility for nesting, which make up ~60 ha combined. With the exception of the water reclamation facility, water levels are not managed by MCBH-KB staff and vary with direct rainfall and tidal fluctuations. The He‘eia National Estuarine Research Reserve is located on the windward side of O‘ahu and is comprised of 120 ha of wetland. Landscape-level restoration efforts are currently underway to restore heterogeneous habitat, such as Hawaiian wetland agro-ecosystems (lo‘i), which provide suitable Hawaiian Stilt nesting habitat (Greer, 2005; Harmon et al., 2020).

Waiawa and Honouliuli wetland units, which are part of the Pearl Harbor National Wildlife Refuge, are situated on the leeward (west) side of O‘ahu and measure 15 ha and 10 ha, respectively. Each wetland is composed of two ponds that receive brackish artesian well water by way of pumps managed by the U.S. Fish and Wildlife Service. Between the 2018 and 2019 Hawaiian Stilt nesting seasons, managers erected a fence around the Honouliuli unit with the purpose of excluding mammalian predators. The fence was in place for the duration of the 2019 and 2020 field seasons. The Ki‘i wetland unit of the James Campbell National Wildlife Refuge, located on the northeastern side of O‘ahu, consists of ~51 ha of stilt nesting habitat and receives freshwater by way of pumps managed by the U.S. Fish and Wildlife Service.

In addition to the mammal exclusion fence at Honouliuli, land managers at each site controlled introduced mammalian predator populations (feral cats, rats, and mongooses) using a combination of New Zealand Department of Conservation 250 traps, Goodnature traps, Tomahawk live traps, and rodenticide bait stations, for trapping and/or removal during the study period.

### Nest surveys

Weekly nest surveys were conducted from February to August in 2018, 2019, and 2020. With the exception of He‘eia, all wetlands were surveyed for a total of two nesting seasons (Table 1). Surveys began with observers driving around the perimeter of the wetland to identify nesting behavior, such as incubation or territorial displays, and then conducting foot surveys to confirm potential nests. All nests were monitored during the incubation period and were considered active if at least one egg was present. For each nest, we recorded GPS coordinates, took measurements of the height of the tallest vegetation within a one m radius of each nest (Ackerman et al., 2014) and recorded the distance from the nest to the nearest water body. All measurements were taken upon first discovery of the nest. In 2019 and 2020, we additionally collected data on whether nests were located on islands, which we defined as surrounded by water in all directions. For a subset of discovered active nests (188 out of 278), a Bushnell No-Glow Aggressor HD Trophy Camera (Bushnell Corporation, Overland Park, KS, USA) was placed ~3 m from the nest, consistent with U.S. Fish and Wildlife Service Threatened and Endangered Species Permitting (#TE-25955C- l), mounted on a 2″ × 1″ furring strip, and secured with a camera strap. Cameras were programed to take two images back-to-back immediately upon infrared motion activation with a 5 s delay between each successive activation. One control photo was taken every hour using field scan mode. Cameras were checked weekly for battery life and data card retrieval and were removed immediately after nests were confirmed as either successful (hatched) or failed. Nests without cameras were checked in person every 3–4 days.

Nests were considered successful if at least one egg hatched and failed if no eggs hatched. Nest fate was determined by observing camera photos, and for nests without cameras, by evaluating habitat conditions within the nest site, which we defined as within one m of the nest. Nests were determined flooded using the following criteria: (1) precipitation data showed a correlation between a recent high intensity rainfall event and time of identified nest failure; and (2) the nest was found underwater and empty, or intact eggs were found outside of the nest following an increase in water level (Bayard & Elphick, 2011). Nests were considered depredated using the following criteria: (1) tracks or feces were observed in the soil, and evidence of bite marks or fragments of eggs were found in or around the nest site; or (2) the nest was found empty prior to the expected hatch date (Cuervo, 2010). Nests were considered abandoned if eggs were intact and present in the nest at 2 weeks or more past the expected hatch date and parents were confirmed to be no longer incubating or defending the nest either by direct observation or by camera photos (Pierce, 1986). While it is possible for nests to become abandoned prior to being depredated, the territorial behavior of stilts, the frequency of our nest surveys, and the use of nest cameras were likely sufficient to determine an inactive nest prior to depredation. Nests were considered hatched if: (1) chicks were observed inside the nest or within the nest site by direct observation or camera photos; or (2) the nest was found empty on or within 1 day of the expected hatch date (Cuervo, 2010; El Malki et al., 2013). This method of determining successful nests accounts for eggshell removal by parents immediately after eggs hatch, as this behavior has been documented for Recurvirostrids (Sordahl, 1994) and was observed during this study via game cameras and direct observations. All methods in this study were permitted by the Institutional Animal Care and Use Committee at the University of Hawai‘i at Mānoa (#17-2733-2), the State of Hawai‘i Department of Land and Natural Resources, Division of Forestry and Wildlife (#WL19-10), and the U.S. Fish and Wildlife Service (#TE-25955C-2).

### Data analyses

All analyses were conducted in the program R 3.5.3 (R Core Development Team, 2019). Linear regression models were used to examine the changes in vegetation height and distance to water at nest sites over the nesting period. In each model we included terms for either vegetation height or proximity to water, along with day of the nesting season. We used a logistic exposure model to predict daily survival probability of nests. Logistic exposure is equivalent to logistic regression with a custom logit link function that accounts for exposure days (Shaffer, 2004). Exposure days for each nest were from the date the nest was found to the date the nest failed or was successful, or to the last date the nest was known to have survived. Dates were scaled such that day one was the first day a nest was found during the nesting season. A set of candidate models were chosen a priori and included terms for day of the nesting season, distance to water, and vegetation height. We used both linear and quadratic terms for day of the nesting season to test for consistent changes in nest survival across the nesting season or a change in nest survival in the middle of the nesting season, respectively. To examine if the effects of distance to water and vegetation height change over the nesting season, we also included models with interactions between these habitat variables and day of the nesting season. Constant daily survival was used as the null model. Candidate models were ranked using Akaike’s Information Criterion corrected for small sample size (AICc), and Akaike’s weights (*w*_*i*_) were used to determine the probability that each model was the best model among the model set. Model comparison results were computed using the *MuMIn* package (Barton, 2016). Before fitting models, we examined the potential effect of nest cameras on nest survival by comparing daily survival between nests with and without cameras and compared the relative support for a camera model to an intercept-only model (constant survival; Richardson, Gardali & Jenkins, 2009). We also compared the relative support for models containing the terms “wetland” and “year” to an intercept-only model to test for differences in nest survival between study sites and years.

Because the use of islands for nesting was only recorded in the 2019 and 2020 nesting seasons, we did not include this variable in our model set; however, we used logistic exposure to compare daily nest survival rates for nests placed on islands and those not placed islands. We also used logsitic exposure to compare daily survial rates of nests in Honouliuli in 2018 with those in Honouliuli in 2019, when the mammal-exclusion fence was complete. To determine if the use of islands and the mammal-exclusion fence are important predictors of nest survival, we compared our logistic exposure models containing these variables to an intercept-only (constant survival) model.

## Results

**Figure 2:**
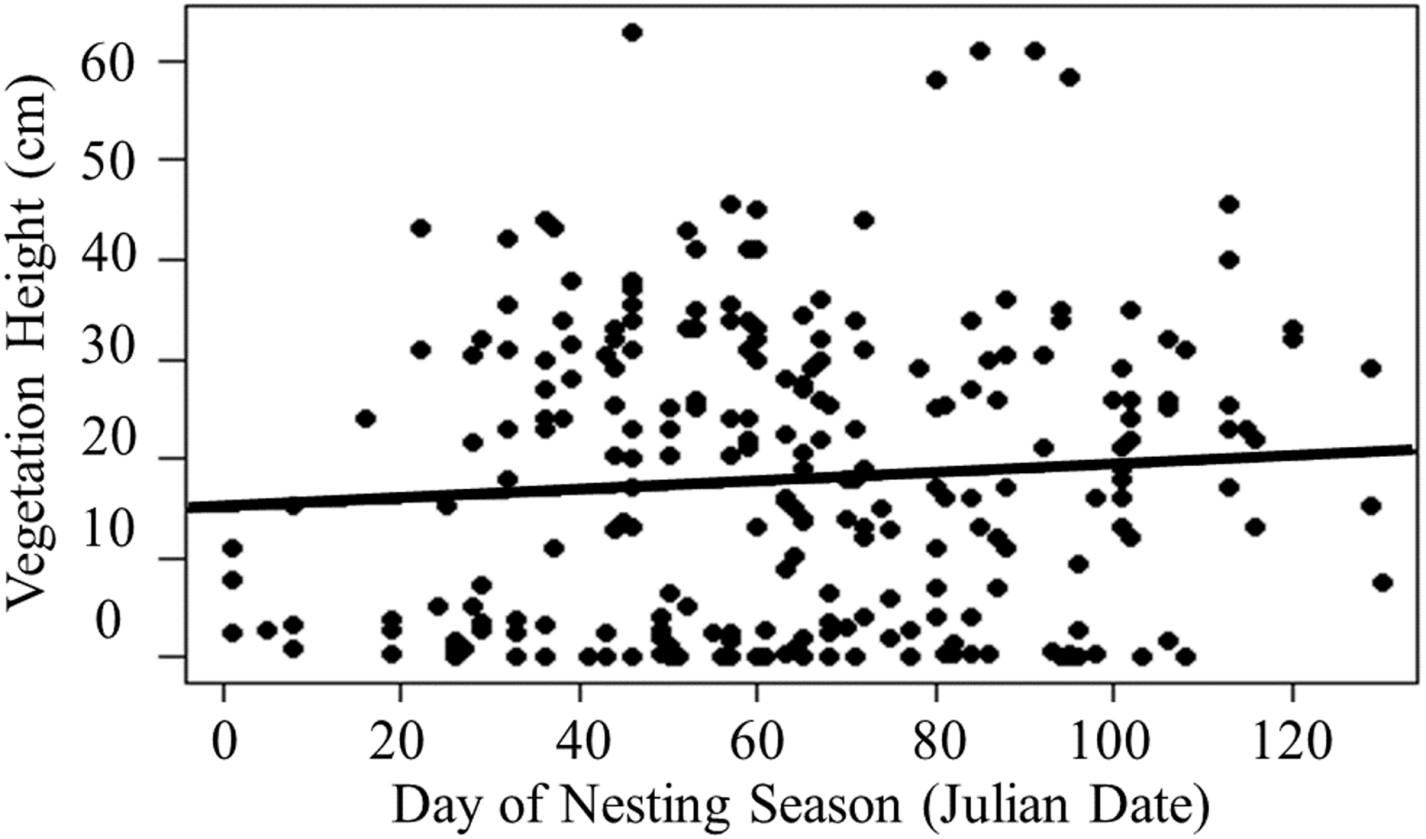
Linear regression of vegetation height at nests across the nesting season. Vegetation height was slightly positively correlated with day of nesting season but did not exhibit a strong linear trend (*R*^2^ = 0.002, *t* = 1.31, *P* = 0.18).

Active nests were discovered from March to July in 2018, from February to July in 2019, and from March to July in 2020. Of 278 nests discovered, 54% (*n* = 149) hatched at least one chick, 17% (*n* = 47) failed due to depredation, 11% (n = 31) failed due to abandonment 3% (*n* = 8) failed due to flooding, and 15% (*n* = 42) had unknown fates. Confirmed egg predators included Small Indian Mongooses (*n* = 7), rats (*n* = 6), feral cats (*n* = 6), Black-crowned Night-Herons (*n* = 4), and Hawaiian Gallinule (*Gallinula galeata sandvicensis*; *n* = 1). We were unable to confirm predator types for 23 depredated nests. Vegetation height at nests ranged from 0 to 63 cm ($\bar x$ = 17.72 cm ± 0.91 SE, *n* = 259), and proximity to water ranged from 0 to 31 m ($\bar x$ = 3.04 m ± 0.26 SE, *n* = 259). While there was a slight positive correlation between vegetation height and day of nesting season, there was not a strong linear trend (*R*^2^ = 0.002, *t* = 1.31, *P* = 0.18; Fig. 2), and proximity to water was not correlated with day of nesting season (*R*^2^ = 0.002, *t* = 0.26, *P* = 0.77).

**Figure 3:**
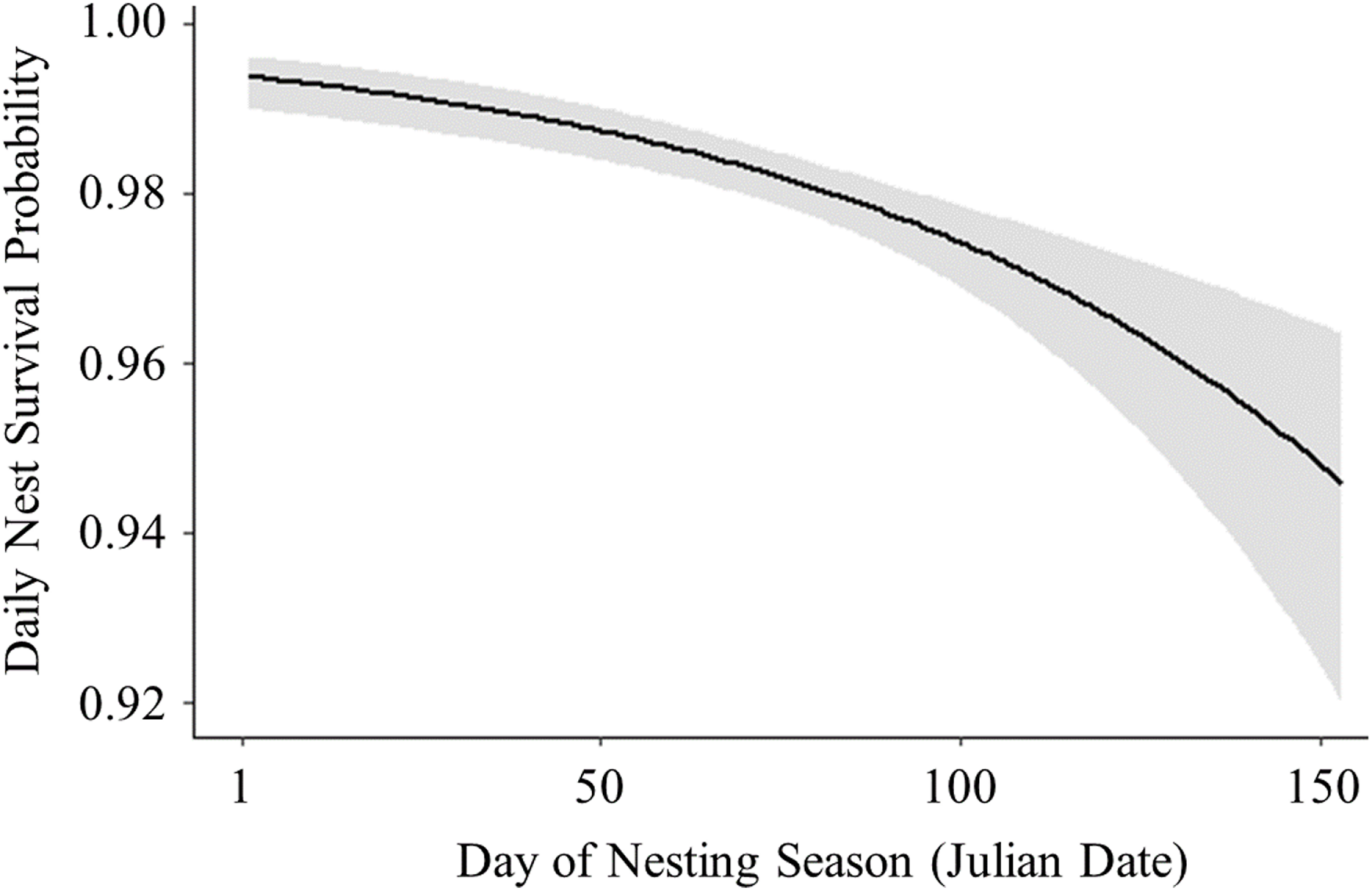
Logistic exposure model showing impact of day of nesting season on nest survival probability. Solid line represents daily survival probability estimated using parameters from the best fit model. Shaded area represents upper and lower 85% confidence intervals for the estimated daily survival probability.

**Figure 4:**
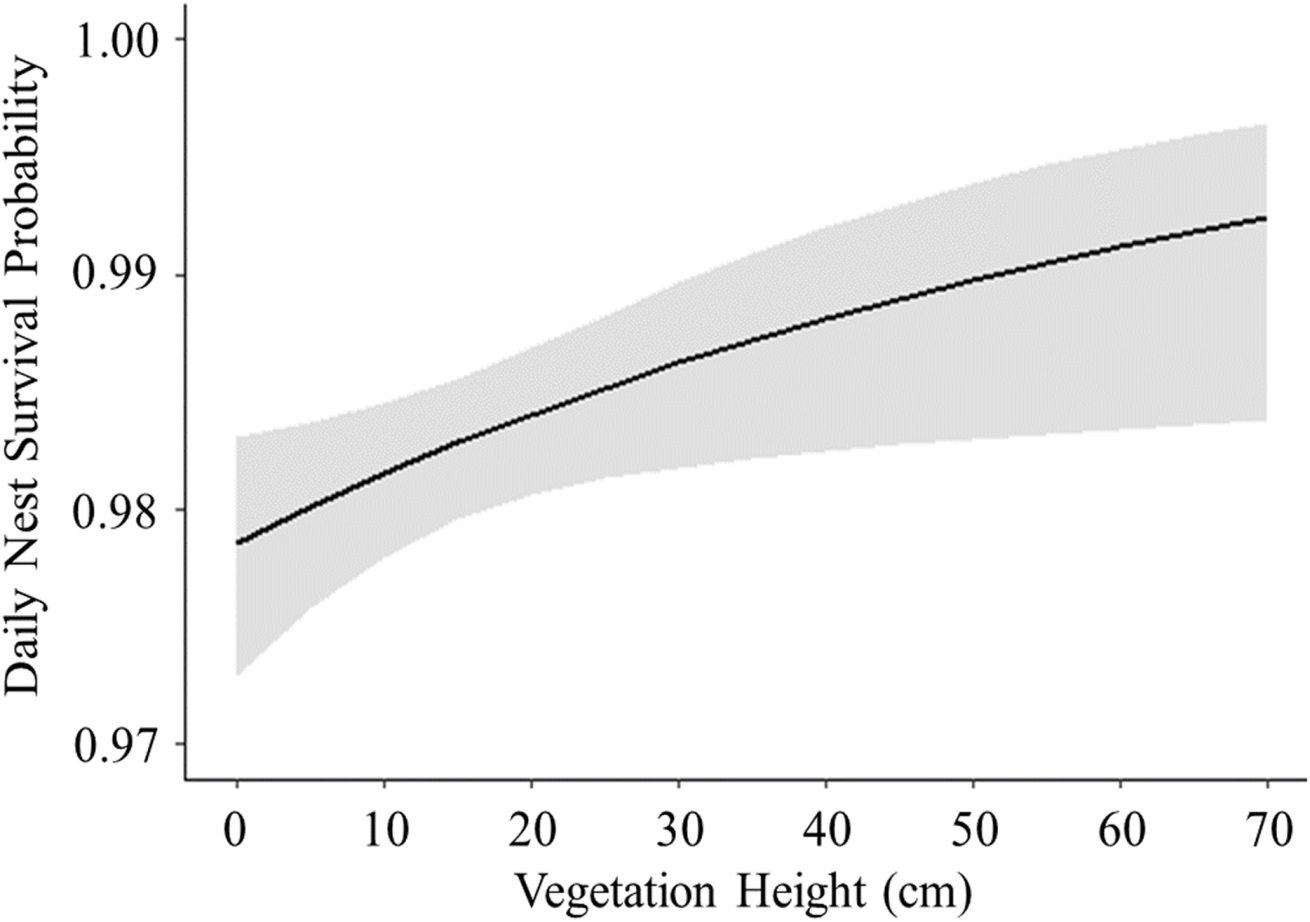
Logistic exposure model showing impact of vegetation height on nest survival probability. Solid line represents daily survival probability estimated using parameters from the best fit model. Shaded area represents upper and lower 85% confidence intervals for the estimated daily survival probability.

**Figure 5:**
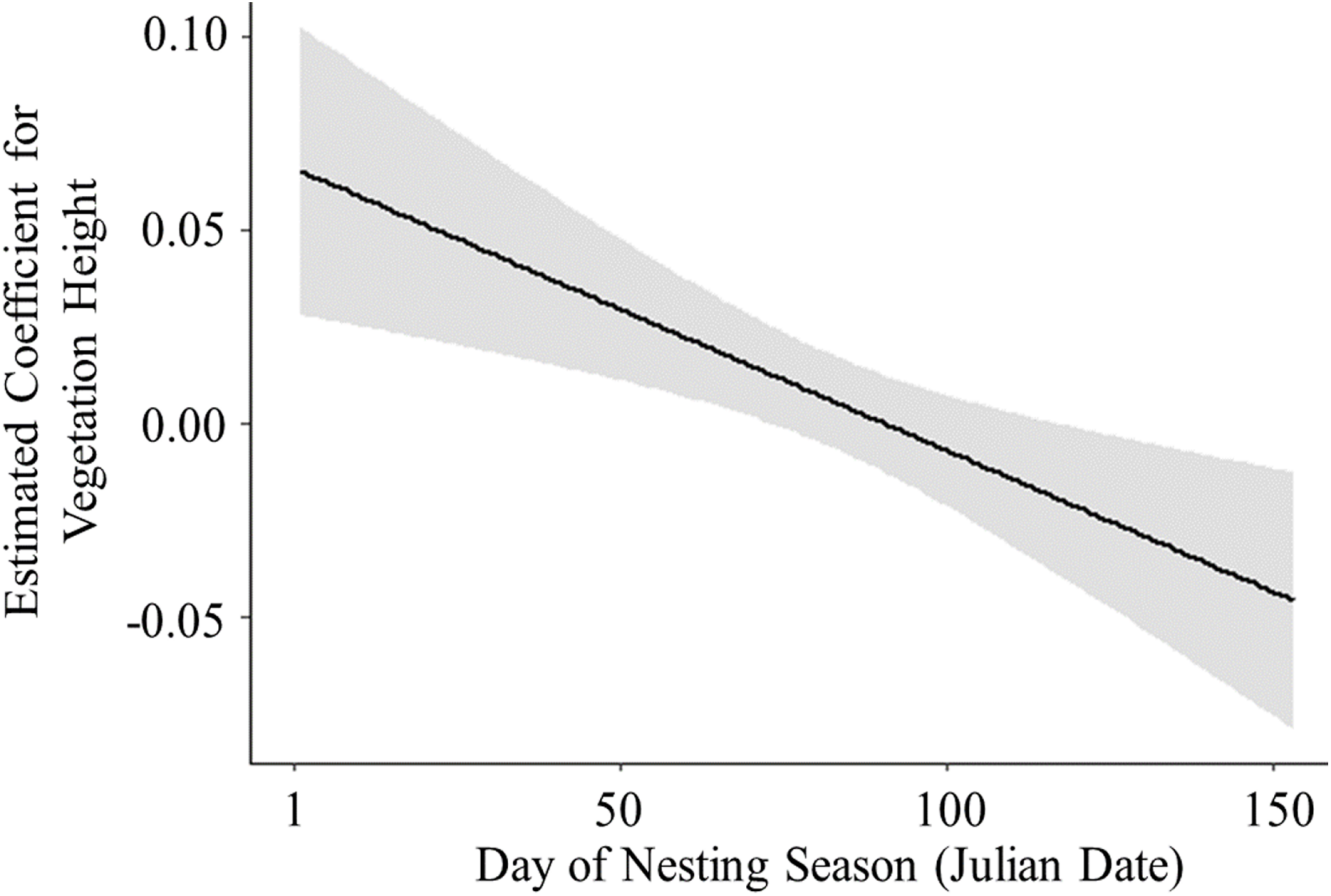
Logistic exposure model showing the estimated coefficient of vegetation height across the nesting season. Solid line represents the slope of vegetation height across the nesting season estimated using parameters from the best fit model. Shaded area represents upper and lower 85% confidence intervals for the slope of vegetation height.

Of 278 total active nests discovered in this study, we were able to use 259 nests in our nest survival analyses; 15 nests were excluded because they were only under observation for 1 day before they failed or hatched, and four nests were excluded because we were unable to collect habitat data. Daily survival rates were similar between nests with cameras ($\bar x$ = 0.97 ± SE 0.003, *n* = 174) and nests without cameras ($\bar x$ = 0.98 ± SE 0.002, *n* = 85), and the presence of cameras was not a significant predictor of nest survival ($X_1^2\;$ = 1.21, *P* = 0.26). Therefore, we did not account for the presence of cameras in our model comparisons (Richardson, Gardali & Jenkins, 2009). There was also little support for models containing the terms “wetland” ($X_6^2\;$ = 7.22, *P* = 0.30) and “year” ($X_2^{2\; }$ = 2.11, *P* = 0.34); as these terms were not the primary interest of this study, they were not included in our model comparison. Of the models compared, the highest ranked model with the lowest AICc value and the largest weight included the linear term for day of nesting season, vegetation height, and their interaction term (Table 2). All models containing a temporal variable had a summed Akaike weight (*w*_*i*_) of 0.98, and models that included the term “vegetation height” had a summed Akaike weight (*w*_*i*_) of 0.70, suggesting strong effects of these variables on nest survival. The top model predicted that daily survival rate decreased with day of the nesting season (Fig. 3) and increased with vegetation height (Fig. 4). The top model also predicted that the effect of vegetation height on nest survival decreased over the nesting season and changed from a positive correlation to a negative correlation a little over halfway through the nesting season (Fig. 5). Models containing the term “proximity to water” had a summed weight of 0.16, providing little support for an effect of this variable on nest survival (Table 2).

**Table 2:** Model selection of Hawaiian Stilt (*Himantopus mexicanus knudseni)* nest survival in wetlands on O‘ahu. Models were ranked using Akaike’s Information Criterion corrected for small sample sizes (AICc). *K* is number of model parameters, ΔAIC is the difference in AIC_c_ value from the top model, and *w_i_* is Akaike model weight.

Model	*K*	AICc	ΔAIC	*w_i_*
Day + Vegetation height + Vegetation height*Day	4	646.00	0.00	0.51
Day	2	648.50	2.51	0.15
Day + Vegetation height	3	649.60	3.60	0.09
Date + Vegetation height + Proximity to water + Vegetation height*Day + Proximity to water*Day	6	649.90	3.88	0.07
Day + Day^2	3	649.90	3.91	0.07
Day + Proximity to water	3	650.40	4.39	0.06
Day + Day^2 + Vegetation height + Proximity to water + Vegetation height*Day + Proximity to water*Day	7	651.90	5.88	0.03
Day + Proximity to water + Proximity to water*Day	4	652.40	6.36	0.02
Null (constant survival)	1	659.30	13.32	0.00
Vegetation height	2	660.90	14.89	0.00
Proximity to water	2	661.10	15.14	0.00
Proximity to water + Vegetation height	3	662.70	16.66	0.00

Daily nest survival rates were similar between nests placed on islands ($\bar x$ = 0.98 ± SE 0.003, *n* = 55) and nests not placed on islands ($\bar x$ = 0.98 ± SE 0.004, *n* = 124), and use of islands was not a significant predictor of nest survival ($X_1^2\;$ = 0.32, *P* = 0.56). In Honouliuli, daily nest survival was lower in 2018 ($\bar x$ = 0.96 ± SE < 0.00, *n* = 24) than in 2019 ($\bar x$ = 1.00 ± SE < 0.00, *n* = 15), and the presence of a mammal-exclusion fence was a significant predictor of nest survival ($X_1^2\;$ = 15.23, *P* < 0.001), suggesting that the mammal-exclusion fence improved nest survival.

## Discussion

This study examined the impacts of seasonality and nest-site characteristics on nest survival of the Hawaiian Stilt in wetlands on O‘ahu. Daily nest survival decreased later in the nesting season, as expected based on other shorebird species in temperate regions (Thyen & Exo, 2005; Cuervo, 2010; Ackerman et al., 2014) and a shorebird species in the tropics (Ramos, 2001). Daily nest survival also increased with increasing vegetation height. This may be due to an increase in nest density (van Rees & Reed, 2020) and thus, competition. If habitat features that are important for nesting success are limited, we would expect the impacts of nest-site characteristics on nest survival to be apparent; thus, if the Hawaiian Stilt population is at or near carrying capacity, competition for suitable nesting habitat may be high. However, there was considerable variation in vegetation height among early and late nests, suggesting that suitable nesting habitat was likely available throughout the nesting season. Further, the effect of vegetation height on nest survival changed from a positive to a negative correlation later in the nesting season. Predator abundance and/or predator trapping effort may have changed throughout the nesting season or alternative prey items of potential predators may have decreased, as these factors were not measured in our study. Predators may have improved their recognition of nests (Nams, 1997) or increased their search intensity in areas where nests were previously found (Tinbergen, Impekoven & Franck, 1967). Additionally, predator types may have shifted during the nesting season, thus altering the importance of tall vegetation. For example, taller vegetation may conceal nests from some predator types, particularly avian species (Kristiansen, 1998; Jedlikowski, Brzeziski & Chibowski, 2015), but may provide cover for ground predators, such as invasive mammals. As Hawaiian Stilts have a variety of nest predators, to better examine the relationships between habitat and Hawaiian Stilt nest depredation, future studies should examine predator-specific impacts (Chiavacci, Bader & Bednarz, 2014).

Alternatively, temporal changes in nest survival may be due to changes in parental behavior, which may affect nest conspicuousness and thus, nest depredation pressure (Cresswell, 1997; Martin, Scott & Menge, 2000; Schmidt & Whelan, 2005). Early nesters may have been of better phenotypic quality and may have been more successful at defending nests from predators (Price, Kirkpatrick & Arnold, 1988; Verhulst & Tinbergen, 1991). Indeed, stilts are highly defensive of nests and chicks (Coleman, 1981; Sordahl, 1996), and in this study, they were frequently observed using anti-predator behavior (broken wing displays) and aggressive flight (“dive-bombing”). As Hawaiian Stilts are able to re-nest if unsuccessful on the first attempt (U.S. Fish & Wildlife Service, 2011), late nests may have been second nesting attempts, and parents may have altered behaviors to account for first-attempt inadequacies; however, we were unable to identify individuals in this study, and thus, could not determine which nests were second attempts.

Proximity to water is often an important predictor of nest survival, as water can deter or attract predators (Picman, 1988; Hoover, 2006; Bonesi & Palazón, 2007) or impact the likelihood of flooding (Polak & Kasprzykowski, 2013). While we did not detect a strong effect of proximity to water on nest survival in this study, and flooded nests only accounted for roughly 9% (*n* = 8) of failed nests, this may have been due to the manipulation of water levels at some sites during our study period. Proximity to water may have also changed at nests throughout the nesting season, further complicating our ability to detect a correlation between water and nest survival. In fact, we often observed water levels changing within days or weeks, correlating with precipitation, temperature, and evaporation rates. Future studies should examine these relationships to inform additional management actions. Furthermore, the use of islands for nesting did not improve daily survival rates. Indeed, depredation was confirmed on nests located on small islands within wetlands and on several occasions, mongooses were observed swimming to nesting areas surrounded by water. Small Indian Mongooses, which were responsible for 30% of confirmed depredation events in this study, have negatively impacted other island avifauna in Hawai‘i (Baker & Russell, 1979), the Caribbean (Seaman & Randall, 1962), Mauritius (Cheke, 1987), and Fiji (Mercer, 1967; Gorman, 1975; Clunie & Morse, 1984). While our study focused on egg survival, nesting in close proximity to water may be important for decreasing chick depredation, as open water does not hold scent and has few obstacles, providing a safe escape route from mammalian predators (Sordahl, 1982). Future studies that include chick survival may help elucidate the impact of nesting near water on Hawaiian Stilt reproductive success.

While most of our conclusions are presented in the context of depredation, as this was the main cause of nest failure in our study, abandonment was the second greatest cause of nest failure and may also be linked to habitat features. As Hawaiian Stilts are territorial during the nesting season, a large number of neighboring nests may cause pairs to abandon their nests (Coleman, 1981). Thus, taller vegetation may have aided in concealing nests, not only from predators, but also from neighboring conspecific pairs. Alternatively, abandonment may be linked to age of nesting pairs (Coleman, 1981), perceived depredation or flooding risk by pairs (Cuervo, 2010), or issues with egg development related to contamination (Herring, Ackerman & Eagles-Smith, 2010). While human disturbance, such as nest visitations by researchers or the use of nest cameras, may also cause nest abandonment in colonial nesting waterbirds (Carney & Sydeman, 1999; Borgmann, 2011), abandonment risk is often greatly reduced when nest checks are limited and conducted during cooler periods of the day (Brown & Morris, 1994), both of which were used in our study. Breeding pairs were always monitored from a distance to check that they returned to the nest after being flushed by researchers, and the majority of abandonment in our study occurred late in the incubation stage, suggesting that factors unrelated to perceived risk (Clutton-Brock, 1991), such as egg development, may have driven abandonment. Further, the use of cameras was not a significant predictor of daily nest survival. More research is needed to investigate the causes of nest abandonment in Hawaiian Stilts.

Our study identified seasonality in nest survival of the Hawaiian Stilt. Our results suggest that impacts of nest-site characteristics on nest survival are likely not constant across the breeding season and may be linked to traits or behaviors of parents, changes in predator activity, or a combination of the two. Our study suggests that subtropical nesting birds may have lower probability of nest survival later in the nesting season, similar to many temperate nesting species, and demonstrates a need to further examine seasonality in nest survival, and its associated factors, in subtropical regions.

## Conclusions

Birds in tropical and subtropical regions generally have smaller clutch sizes, longer chick development times, longer nesting seasons, and more nesting attempts than birds in temperate regions (Skutch, 1985; Martin, 1996; Ricklefs, 1968, 1976). Thus, within-season timing of nesting may not be as important for nesting success of birds nesting in tropical and subtropical regions. To date, few studies have examined seasonality of nest survival in tropical and subtropical species (Morrison, 1999; Olmos & Silva, 2001; Ramos, 2001). This study identified seasonal patterns in nest survival of the Hawaiian Stilt, a subtropical wading bird. Early nesters had higher survival probability than late nesters. The importance of vegetation for nest survival was dependent on day of the nesting season, and proximity to water was not found to impact nest survival. However, temporal patterns in nest survival may also be attributed to parental behavior or predator activity. While we were able to examine impacts of some habitat characteristics, additional studies may further elucidate the drivers of seasonal variation in nesting success of the Hawaiian Stilt. Furthermore, given the projected impacts of climate change on tropical and subtropical shorebird habitat (Amano et al., 2020; Xi et al., 2020; van Rees & Reed, 2018; K.C. Harmon, 2020, unpublished data). This study has important implications for other shorebirds nesting in tropical and subtropical habitats and suggests the need for more research that examines seasonality in shorebird nesting success in these regions.

## Supplemental Information

10.7717/peerj.10399/supp-1 Supplemental Information 1 Season patterns in nest survival of the Hawaiian Stilt - Data Analyses. R script containing code to run all analyses

10.7717/peerj.10399/supp-2 Supplemental Information 2 Season patterns in nest survival of the Hawaiian Stilt - Dataset. Dataset used for all analyses

## Additional Information and Declarations

### Field Study Permissions

The following information was supplied relating to field study approvals (i.e., approving body and any reference numbers): Field surveys were permitted by the Hawai‘i State Department of Land and Natural Resources, Division of Forestry and Wildlife (permit #WL19-10), as well as the U.S. Fish and Wildlife Service (permit #TE-25955C-2).

### Data Availability

The following information was supplied regarding data availability: Raw data and code are available in the Supplemental Files.
